# Asymmetries in Accessing Vowel Representations Are Driven by Phonological and Acoustic Properties: Neural and Behavioral Evidence From Natural German Minimal Pairs

**DOI:** 10.3389/fnhum.2021.612345

**Published:** 2021-02-18

**Authors:** Miriam Riedinger, Arne Nagels, Alexander Werth, Mathias Scharinger

**Affiliations:** ^1^Department of English and Linguistics, Johannes Gutenberg University, Mainz, Germany; ^2^Institute for German Linguistics, Philipps University, Marburg, Germany; ^3^Department of Language and Literature, Max Planck Institute for Empirical Aesthetics, Frankfurt, Germany

**Keywords:** vowel discrimination, mismatch negativity (MMN), reaction time (RT), multiple regression analysis, perceived loudness

## Abstract

In vowel discrimination, commonly found discrimination patterns are directional asymmetries where discrimination is faster (or easier) if differing vowels are presented in a certain sequence compared to the reversed sequence. Different models of speech sound processing try to account for these asymmetries based on either phonetic or phonological properties. In this study, we tested and compared two of those often-discussed models, namely the Featurally Underspecified Lexicon (FUL) model (Lahiri and Reetz, 2002) and the Natural Referent Vowel (NRV) framework (Polka and Bohn, 2011). While most studies presented isolated vowels, we investigated a large stimulus set of German vowels in a more naturalistic setting within minimal pairs. We conducted an mismatch negativity (MMN) study in a passive and a reaction time study in an active oddball paradigm. In both data sets, we found directional asymmetries that can be explained by either phonological or phonetic theories. While behaviorally, the vowel discrimination was based on phonological properties, both tested models failed to explain the found neural patterns comprehensively. Therefore, we additionally examined the influence of a variety of articulatory, acoustical, and lexical factors (e.g., formant structure, intensity, duration, and frequency of occurrence) but also the influence of factors beyond the well-known (perceived loudness of vowels, degree of openness) in depth *via* multiple regression analyses. The analyses revealed that the perceptual factor of perceived loudness has a greater impact than considered in the literature and should be taken stronger into consideration when analyzing preattentive natural vowel processing.

## Introduction

In recent years, much research has been done on the mental representations of vowels and on investigating which properties are involved in vowel discrimination. This article investigates the mental representations of vowels and compares two models that both make specific hypotheses regarding sound discrimination and mental representations of speech sounds, namely the Featurally Underspecified Lexicon (FUL) model (Lahiri and Reetz, 2002, 2010) and the Natural Referent Vowel (NRV) framework (Polka and Bohn, 2011). Based on the notions that spoken language has a sequential, serial structure and those earlier events that precede later events influence the recognition or discrimination of those later events, both models have in common that they are predicting directional asymmetries in the discrimination of speech sounds: discrimination of two speech sounds is easier in one direction than in the other; therefore, it matters which sound is presented first. For example, when testing the discrimination of two vowels (e.g., [i] and [e]), one can present the vowels in two possible orders: the high vowel followed by the mid vowel ([i]—[e]) or in the reverse order ([e]—[i]). Both models assume that vowel discrimination is based on the nature of the mental representation and predict facilitated discrimination in one direction, but predictions about the easier presentation order are often competing. Furthermore, what separates the models are the substantially different assumptions about the features involved in discrimination processes and therefore in mental representations.

Within the FUL model, Lahiri and Reetz (2002, 2010) made a proposition for speech perception and lexical access suggesting that speech sounds can be described with the help of abstract and underspecified feature specifications (e.g., [HIGH] for high or close vowels, such as [i]). Importantly, they also describe sound processing based on those features. Crucially, this model assumes that there can be a discrepancy between the features contained in the signal and those stored in the mental lexicon, since mental representations may be underspecified and therefore do not contain all possible features. These assumptions of underspecified mental representations express both similarities and differences to other approaches of underspecification. In common with other underspecification theories, the underspecified sound descriptions are based on the notion of *minimalism*. In this respect, it is postulated that only a distinct set of sound descriptors are necessary for underlying representations. But in contrast to theories like Radical Underspecification (Archangeli, 1988) the underspecification approach in FUL is not only a theoretical means to describe certain linguistic phenomena (e.g., assimilation) but also constitutes mental representations of speech. Therefore underspecification is directly involved in speech perception and production. Additionally, in FUL sounds can be described solely with monovalent features. For example, in FUL it is believed that coronal segments (i.e., front vowels) are underspecified for a place of articulation information ([–]) in the mental representation, but the feature [COR] can be retrieved from the auditory signal. This underspecification approach, together with the specific proposed ternary mapping process, is the reason for the resulting directional asymmetries in sound discrimination. This mapping process includes a comparison of the features obtained from the signal with those stored in the mental lexicon. Due to the underspecification of redundant features, there are three possible outcomes: a match occurs if the feature extracted from the signal has the same equivalent feature in the mental lexicon (e.g., [DOR]—[DOR]: [u]—[o]). A mismatch occurs if the feature taken from the signal and the feature in the underlying representation are complementary and exclude each other (e.g., [HIGH]—[LOW]: [i]—[a]). Last but not least, a no-mismatch occurs if a feature extracted from the signal neither mismatches with a feature of the mental lexicon nor matches it. The last setup of the mapping process is crucial in the elicitation of directional asymmetries. For example, if [COR] is extracted from the signal, this feature produces a mismatch with [DOR] in the lexicon, but if [DOR] is extracted from the signal, the result is a no-mismatch due to the underspecification of the coronal place of articulation ([–]). These different results should become apparent when the discrimination of two vowels is tested in both possible presentation orders ([i]—[u] vs. [u]—[i]).

Several studies have shown that the presentation order with a mismatch as the result of the mapping process usually elicits larger effects than vice versa. Eulitz and Lahiri (2004) conducted an ERP study with German vowels [o], [ø], and [e], which differ mainly in place of articulation. When discriminating [o]—[ø], larger electrophysiological responses occurred because of the mismatching features [DOR]—[COR]. In the reverse direction, the effects were attenuated due to the underspecification of the coronal place of articulation. Similar results have been produced by Scharinger et al. (2012b) for tongue height oppositions using American English vowels for which the mid of tongue height is believed to be underspecified. They found larger effects if the mid vowel [ε] had to be discriminated from low vowel [æ] due to the mismatching features of [LOW] and [MID] compared to the reverse sequence, in which there is no feature mismatch due to underspecification. Similar evidence for this approach has been found not only for vowels (Lipski et al., 2007; de Jonge and Boersma, 2015) but also for consonants (Hestvik and Durvasula, 2016; Schluter et al., 2016, 2017; Cummings et al., 2017; Højlund et al., 2019; Hestvik et al., 2020) and suprasegmental elements like lexical tones (Politzer-Ahles et al., 2016). While most studies used isolated vowels or syllables, there is also evidence from complex stimuli like words (Friedrich et al., 2008; Scharinger et al., 2012b; Cornell et al., 2013; Lawyer and Corina, 2018).

The other model investigated in this article, the NRV framework, also predicts different discrimination performances as a function of presentation order. In contrast to the aforementioned model, NRV operationalizes phonetic properties of the speech signal which can be specified by acoustical or visual cues to explain directional asymmetries and predict different discrimination performances and proposes that “vowels with extreme articulatory-acoustic properties (peripheral in the vowel space;” Polka and Bohn, 2011, p. 474) are so-called referent vowels and are easier to discriminate. Polka and Bohn (2003, 2011) observed a universal perceptual bias favoring vowel discrimination from a more central to a more peripheral vowel in the vowel space in infants. They proposed that the vowels on the periphery of the vowel space (/i/, /a/, /u/, /y/) act as universal referent vowels in language development and vowel discrimination due to their more salient and extreme articulatory-acoustic properties. The vowel space periphery’s perceptual advantage can be explained by the convergence of adjacent formants and therefore the stronger focalization of the referent vowels (Schwartz et al., 1997, 2005). Since this framework has been developed from the point of view of language acquisition and infant vowel discrimination, much work has been done on the investigation of the proposed perceptual bias in infants. There is evidence from an early cross-linguistic study with German- and English-learning infants that for English vowels /ε/ and /æ/, discrimination was easier for /ε/—/æ/ than in the reverse direction, regardless of the language background of the infants (Polka and Bohn, 1996). A similar bias with easier discrimination from a more central (less focal) to a more peripheral (more focal) vowel was shown in several studies (Bohn and Polka, 2001; Polka and Bohn, 2011; Pons et al., 2012; Simon et al., 2014). Additionally, there is some encouraging evidence that the perceptual bias preferring some sounds to others in discrimination in infants also could hold true in consonants (Nam and Polka, 2016). Concerning adult vowel perception and discrimination, within the framework, it was initially proposed that the perceptual bias is shaped by language experience. Therefore, the asymmetry only occurs if subjects are discriminating non-native vowel contrasts, while in native vowel contrasts, the perceptual bias disappears, and asymmetry occurs (Polka and Bohn, 2011). The assumption of experience-dependent asymmetries was found in some studies (Tyler et al., 2014; Kriengwatana and Escudero, 2017) while others also report universal biases in adults. In an AX discrimination test with Canadian-French and Canadian-English subjects using tokens of less focal English /u/ and more focal French /u/, Masapollo et al. (2017b) found that discrimination from less to more focalization produced better and faster results irrespective of language background. Therefore, the authors argued that there is a universal bias towards more focalized vowels in adults, too. These results have been replicated and extended in that the universal bias seems to have an impact not only on the auditory domain of speech processing but also on visual vowel discrimination (Masapollo et al., 2017a, 2018).

In recent research, mental representations of speech sounds have often been investigated with the help of electrophysiological methods, for example by using event-related potentials (ERPs). ERPs offer a means to investigate speech processing on a temporal axis with the accuracy of milliseconds. In the investigation of speech sound processing, one ERP component, the so-called Mismatch Negativity (MMN), has become prominent. The MMN can be defined as a specific electrophysiological detection change response of the brain when the repetitive presentation of one stimulus (standard) is interrupted occasionally and unpredictably by a different stimulus (Näätänen et al., 2007). The MMN component has often been used for the investigation of (speech) sound processing since this component can be elicited even when participants are not attending to the stimulation. It, therefore, reflects preattentive and automatic speech processing, making it possible to differentiate the neural responses of stimuli without attention effects and other perceptual and cognitive processes (for a review on the component, see Näätänen et al., 2007). This component usually peaks fronto-centrally between 100 and 250 ms after change onset and can be elicited by any discriminable change in the stimulation (Näätänen, 2001), for example in pure tones (e.g., Sams et al., 1985), with sensitivity for changes in frequency (for example Takegata and Morotomi, 1999; Tervaniemi et al., 2000), intensity and duration (e.g., Paavilainen et al., 1991) but also in more complex stimuli like speech sounds (Dehaene-Lambertz, 1997; Dehaene-Lambertz et al., 2000). Furthermore, several studies have shown that the latency of the component is usually linked to the complexity of the stimuli, while the amplitude of the MMN is correlated with the magnitude of deviation. The greater the differences between the standard and the deviant stimulus are, the greater the MMN (e.g., Sams et al., 1985; Savela et al., 2003). Moreover, it has been shown that the MMN component is sensitive for language-specific phonemic processing of speech sounds (e.g., Dehaene-Lambertz, 1997; Näätänen et al., 1997), which led to the interpretation that mental representations are of phonemic or phonetic nature (as opposed to auditory ones) and language-specific.

In this article, we tested both competing models with German vowels to investigate which model can best explain directional asymmetries. Consequently, we tested the predictions of both models on a large stimulus set (five German long vowel contrasts) in a more natural listening situation by using real minimal pairs. The study was based on the following notions.

The use of real words in MMN investigations is associated with obstacles due to interference, such as lexical status, familiarity, or other confounding factors. In several studies, it has been shown that MMN responses for real word deviants are enhanced in comparison to pseudowords: it is believed that the enhancement of the lexical MMN is due to stronger memory trace activation for real meaningful words (Pulvermüller et al., 2001, 2004; Shtyrov and Pulvermüller, 2002; Endrass et al., 2004; Pettigrew et al., 2004a; Shtyrov et al., 2008). Another known influential factor on speech processing is the lexical frequency of the used real words. This influence can even be present when testing real words in a passive oddball paradigm and can lead to a stronger MMN response for words with higher lexical frequency in opposition to deviants with a lower or intermediate frequency of occurrence (Alexandrov et al., 2011; Shtyrov et al., 2011; Aleksandrov et al., 2017). Furthermore, it has been shown that phonotactic probabilities (sequential order of phonemes in words) influence MMN results with higher probability, accompanied by enhanced MMN effects (Bonte et al., 2005; Yasin, 2007; Emmendorfer et al., 2020). Concerning vowel perception, acoustic properties, like for example fundamental frequency, vowel duration or intensity (Aaltonen et al., 1994; Kirmse et al., 2007; Peter et al., 2010; Partanen et al., 2011), have an impact on neural effects. While some of the mentioned influential factors can be controlled for when developing stimulus materials, others are not avoidable. For instance, various acoustic differences in vowels stem from collinearities between vowel identity and acoustic consequences. Changes in vowel identity simultaneously lead to changes in the spectral frequency structure (mainly F1 and F2) of the stimuli. Moreover, vowel features used in theoretical frameworks are based largely on articulatory-acoustic properties—mainly formants—and therefore, it could also be possible that there is more of an acoustical influence, especially in MMN effects, than proposed by operationalizing more abstract and theoretical derived features. For instance, the feature opposition of [HIGH] and [LOW] is the more abstract representation of the articulatory and acoustic properties of those vowels concerning the first formant: high vowels have a low F1, while low vowels have a high F1 (Lahiri and Reetz, 2010). Also, the abstract description of vowels referring to focality which were used in the NRV framework is based on articulatory-acoustic properties, since focalization stems from the convergence of adjacent formants (Schwartz et al., 1997). While the common contributing articulatory-based factors of vowel perception have often been investigated, the influence of perceptual and psychoacoustic parameters (e.g., perceptual loudness) on vowel perception has hardly been studied. Thus, we wanted to additionally investigate which were the influential factors on vowel discrimination, including not only theoretical and acoustical factors but also perceptual factors beyond the well-known.

Hence, the following research questions shall be investigated: (1) which model accommodates directional asymmetries in the processing of natural and unmanipulated German long vowels in the best way; and (2) which factors influence vowel discrimination in natural German minimal pairs? The first question has been addressed on an electrophysiological level through measurement of MMN (Experiment 1) and on a behavioral level in means of reaction times (RT; Experiment 2). The second aim of identifying influential factors on vowel discrimination, pursued *via* multiple regressions on both datasets, should shed more light on factors that co-determine MMN effects.

## Experiment 1: Mmn Study

To test both models, we first conducted an MMN study with a large stimulus set, testing five German long vowel contrasts embedded in natural minimal pairs, which almost mapped the entire German (long) vowel space. The vowels chosen for investigation were among the most frequent long vowels in the German language (Aichert et al., 2005).

### Participants

Nineteen participants (nine females, mean age 24.7, SD 3.4), graduate and undergraduate students of the Philipps University of Marburg, participated in two sessions for monetary compensation. They were all right-handed and reported no hearing or neurological impairments. All participants were monolingual German native and German Standard speakers without being able to speak any German dialect actively. They were all born and socialized in Hesse, Germany, with Standard German. The information about the participants’ dialect and Standard German competence was retrieved by questionnaire. Informed written consent was obtained from each participant before the experiment. One subject had to be excluded because the participant missed the second session. Another subject had to be excluded due to excessive contamination with artifacts in the EEG data (movement artifacts). In total, we assessed and analyzed the complete data of 17 participants.

### Materials

To test the hypotheses of the aforementioned models, we chose the five German long vowel contrasts /i:/—/e:/, /e:/—/a:/, /y:/—/u:/, /i:/—/u:/, and /i:/—/a:/. They differed concerning the place of articulation, vowel height as well as rounding. To ensure more phonological processing, we embedded these vowels in German monosyllabic minimal pairs. We tried to keep the phonetic context between pairs as similar as possible. We also controlled for the frequency of occurrence with SUBTlex (Brysbaert et al., 2011), as seen in Table 1.

**Table 1:** Phonetic and lexical parameters of the vowels.

Vowel contrast	Words F0 (SD)	Mean F1 (SD)	Mean F2 (SD)	Mean F3 (SD)	Mean intensity (SD)	Mean duration (ms)	Mean (log-values)	Word frequency
/a:/—/i:/	*Zahl* (“number”)	169 (24)	875 (19)	1,488 (59)	3,046 (77)	72.85 (0.27)	292	2.861
	*Ziel* (“target”)	203 (5)	244 (28)	2,445 (61)	3,446 (74)	73.79 (0.92)	159	3.358
/e:/—/i:/	*Steg* (“bridge”)	179 (10)	359 (6)	2,483 (58)	3,171 (113)	73,96 (0.67)	270	1.255
	Stieg (“climbed”)	190 (3)	286 (11)	2,479 (51)	3,528 (21)	74.92 (0.31)	228	2.352
/a:/—/e:/	*Mahl* (“meal”)	174 (5)	913 (10)	1,484 (47)	2,966 (44)	71.68 (0.22)	225	1.672
	*Mehl* (“flour”)	191 (3)	341 (8)	2,566 (39)	3,377 (209)	71.74 (0.26)	195	1.857
/u:/—/i:/	*Stuhl* (“chair”)	200 (8)	294 (37)	1,974 (212)	2,647 (99)	73.88 (0.44)	186	2.892
	*Stiel* (“handle”)	199 (6)	275 (22)	2,475 (75)	3,579 (59)	73.67 (0.44)	176	1.756
/u:/—/y:/	*Sud* (“brew”)	194 (2)	296 (40)	1,145 (140)	2,628 (93)	74.75 (0.20)	194	0.845
	*Süd* (“south”)	180 (5)	292 (14)	2,085 (120)	2,633 (146)	74.43 (0.43)	223	1.771

*Mean F0, F1, F2, and F3 values are given in Hertz for vowels per word category. Mean Intensity for the vowels within the words are given in dB. Mean duration measures referring to the vowels within the words. The frequency of occurrence (as log-values) for each word is given in the last column*.

**Figure 1:**
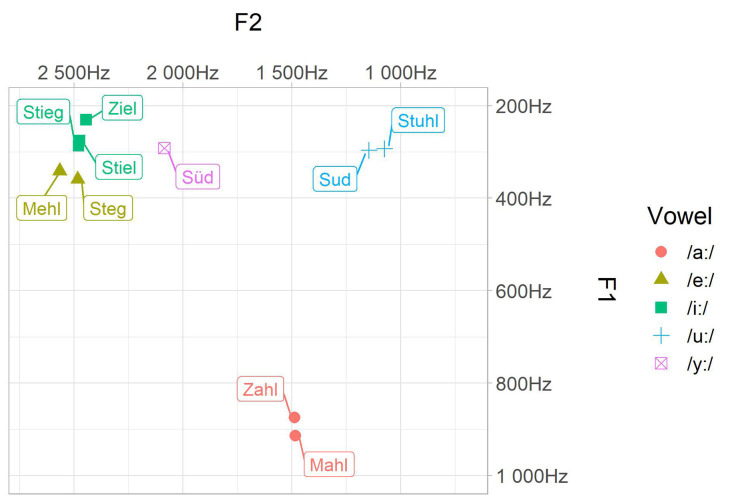
Acoustic characteristic of the stimuli. Mean values of the first (F1) and the second (F2) formant are given per word category in Hertz.

Twenty natural exemplars of each word were recorded in a sound shielded booth by a female German Standard speaker who was phonetically trained. All tokens were spoken with neutral pronunciation. All sounds have been analyzed for F0, F1, F2, F3, as well as vowel duration and were all scaled to an intensity level of 70 dB within Praat (Boersma and Weenink, 2016). The five best tokens per word have been chosen as experimental stimuli. Phonetic parameters of the word categories are displayed in Table 1. Note that we reported here only mean values per word category (since MMN and RT data are also averaged measures), but a more detailed description of the acoustic parameters can be found in Supplementary Table 1. There can be seen that our stimuli had some variance regarding, for example, vowel duration. Since, we wanted to test natural spoken words, no manipulation was applied. All vowels should be perceived as long vowels despite the length differences since the phonological category is additionally supported by the lexical context. Therefore, the focus in processing lies on categorical differences regarding vowel height and place of articulation. All experimental stimuli were found to sound natural by two different persons. All tokens were also assessed as being distinct for their category (see Figure 1). We compared the formant values (F1, F2) to the ones of Sendlmeier and Seebode (2006) to ensure that they will be perceived as Standard German. We chose to introduce inter-token variation to obtain a more natural listening situation and to ensure a more phonological approach since participants are forced to map the incoming variable acoustic signals onto a unified and more abstract representation to cope with inter-token variability (Phillips et al., 2000; Eulitz and Lahiri, 2004; Jacobsen et al., 2004). This is an important design feature since it mediates the likely collinearity between formant frequencies and acoustic- or articulatory-phonetic features.

### Task and Procedure

The stimuli were embedded in a passive oddball design. In this paradigm, the participants were presented with a series of repetitive stimuli (standards) that were interspersed occasionally by a deviant varying only in vowel quality while they were watching a silent movie. The frequently presented standards were assumed to activate the memory trace and therefore the representation in the mental lexicon, whereas the infrequently presented deviants provided information about and are processed closer to the surface structure. Each vowel contrast was tested bidirectionally. Because, we investigated five contrasts in both directions, all subjects were tested in two sessions (with testing times per session approximating 2 h) within 15–20 days. Thus, each word served as standard and as deviant in different blocks and sessions.

Each contrast direction was presented in two blocks containing 425 standards and 75 deviants each. In total, we presented 850 standards and 150 deviants per contrast direction. Thus, we presented 2,000 stimuli for each vowel contrast. Within the blocks, stimuli were randomized, and the interval between two deviants randomly consisted of 4–11 standards. Blocks were randomized for both sessions. Blocks of the same condition never succeeded each other. The fixed ISI was 1,000 ms, while the stimuli varied in duration. Therefore, we still obtained a jittered presentation to suppress rhythmic processing and habituation to synchronously presented stimuli.

Subjects were seated comfortably in a sound-insulated and electromagnetically shielded chamber in front of a screen. Sounds were presented binaurally at a comfortable listening level *via* two loudspeakers on the left and the right of the screen, using the open-source software OpenSesame (Mathôt et al., 2012). The listening level was set before the experiment and was kept equal across all subjects (based on the intensity level of 70 dB as manipulated in PRAAT).

### Hypotheses

Since this article aims at comparing the two aforementioned models, hypotheses have been made on the assumptions of vowel discrimination following FUL as well as NRV. For NRV we proposed the hypothesis based on the universal assumptions of the framework that vowels /i:/, /y:/, /u:/, and /a:/ are reference vowels (Polka and Bohn, 2011). The basic assumptions of both models regarding feature specifications and position in the vowel space for each investigated contrast are displayed in Table 2.

**Table 2:** Assumptions for feature specifications (FUL) and location in the vowel space (NRV) for each vowel contrast and hypotheses for mismatch negativity (MMN) effects according to both models.

Vowel contrast	Presentation order	Features (FUL)	Mapping result (FUL)	Expectations MMN (FUL)	Classification (NRV)	Expectations MMN (NRV)
/a:/—/i:/	*Zahl—Ziel*	[DOR]—[COR] [LOW]—[HIGH]	Mismatch Mismatch	Stronger effect	Both peripheral	Symmetrical effect
	*Ziel—Zahl*	[–]—[DOR] [HIGH]—[LOW]	No-mismatch Mismatch	Weaker effect		
/e:/—/i:/	*Steg—Stieg*	[–]—[HIGH]	No-mismatch	Weaker effect	Central—peripheral	Stronger effect
	*Stieg—Steg*	[HIGH]—[MID]	Mismatch	Stronger effect	Peripheral—central	Weaker effect
/a:/—/e:/	*Mahl—Mehl*	[DOR]—[COR] [LOW]—[MID]	Mismatch Mismatch	Stronger effect	Peripheral—central	Weaker effect
	*Mehl—Mahl*	[–]—[DOR] [–]—[LOW]	No-mismatch No-mismatch	Weaker effect	Central—peripheral	Stronger effect
/u:/—/i:/	*Stuhl—Stiel*	[DOR]—[COR]	Mismatch	Stronger effect	Both peripheral	Symmetrical effect
	*Stiel—Stuhl*	[–]—[DOR]	No-mismatch	Weaker effect		
/u:/—/y:/	*Sud—Süd*	[DOR]—[COR]	Mismatch	Stronger effect	Both peripheral	Symmetrical effect
	*Süd—Sud*	[–]—[DOR]	No-mismatch	Weaker effect		

In accordance with the models, we predict the following MMN effects (see also Table 2): within FUL, the effects should be stronger for mismatching presentation orders /a:/—/i:/, /u:/—/y:/ and /u:/—/i:/ (because of the mismatching features [DOR] and [COR]), /i:/—/e:/ (due to mismatch of [HIGH] and [MID]) as well as /a:/—/e:/ (mismatch of [DOR]—[COR] and [HIGH]—[MID]). If NRV holds true, MMN effects should be stronger when *Stieg* and *Mahl* are deviants since they are referent vowels in this models. In the other three contrasts, a symmetry should occur since both vowels are peripheral and act as referents within the framework.

### EEG Recording and Analysis

EEG was recorded with 28 Ag/AgCl passive electrodes connected to a BrainAmp amplifier (Brain Products GmbH). Electrodes were arranged on an EasyCap in 10-20 positions. AFz served as the Ground electrode, and the online reference was placed on the nose tip. Four additional electrodes measured the electrooculogram (EOG) for the identification of artifacts caused by eye movements (e.g., blinks). Two electrodes were placed left and right of the eye canthi to measure lateral eye movements. Two electrodes above and under the right eye measured vertical eye movements. For all electrodes, impedances were kept below 5 kΩ and the sampling rate was 500 Hz.

EEG analysis was done with the MATLAB toolbox fieldtrip. Raw data were filtered with 0.16 and 30 Hz high- and low-pass filters. Data were re-referenced offline to linked mastoids. After segmentation, EEG data were automatically corrected for muscle artifacts. Eye movements were automatically corrected through the correlation of EOG channels and ICA components. The calculation of the MMN component was based on the onset of the vowel, i.e., epochs beginnings were aligned with vowel beginnings. Thereby, consonant onset clusters in the stimuli should play no role in the MMN effects. Additionally, ERP data were baseline corrected using the 100-ms prestimulus epoch.

For averaging the first ten standards of a block and the first standard after a deviant were excluded from data analysis. To maintain ERP results without the influence of pure acoustic influences, we calculated and plotted the MMN as identity MMN (iMMN). Here, the standard and the deviant of the same word are compared to each other (Pulvermüller et al., 2006).

### Results

**Figure 2:**
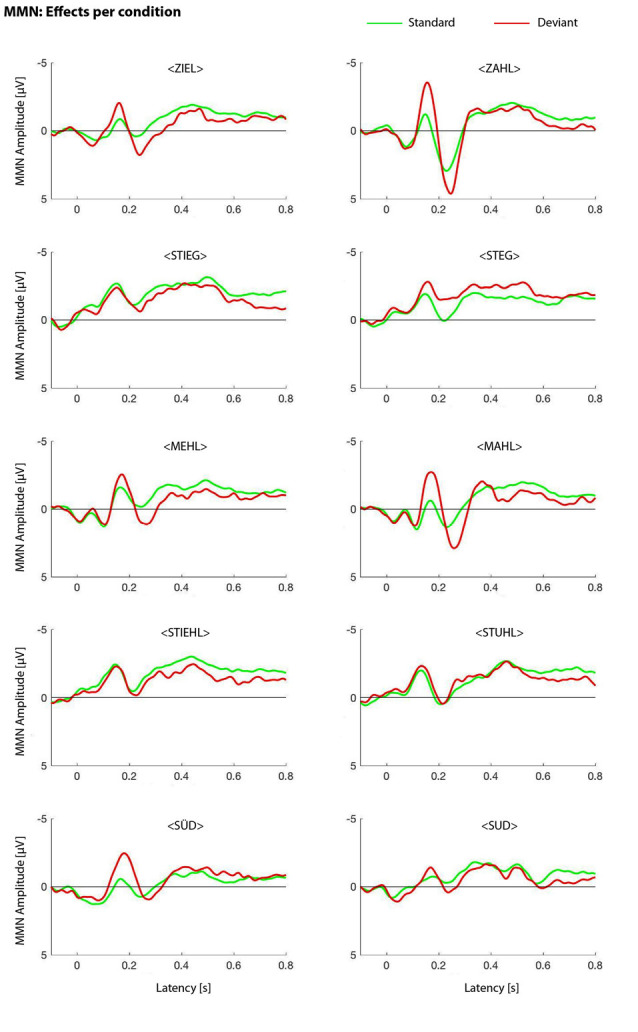
Identity mismatch negativity (MMN) effects per condition. MMN waveforms for all word pairs, in both presentation orders, are shown.

The results of the iMMN study are plotted in Figure 2.

**Figure 3:**
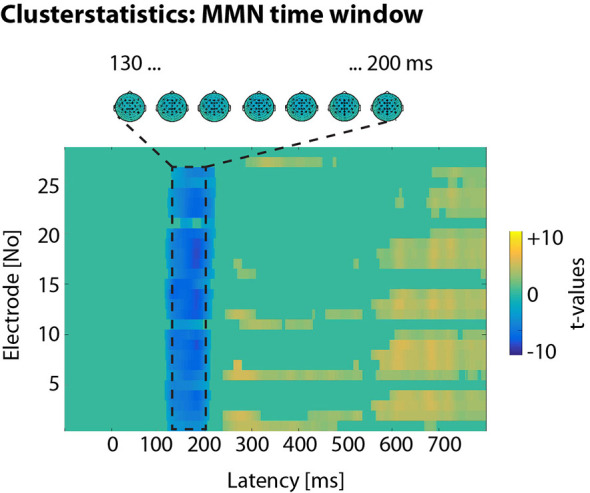
Clusterstatistics. In an electrode × time cluster, deviants elicited more negative responses than standards in the time window between 130 and 200 ms post vowel onset.

In a first step, we were interested in significant standard-deviant differences in the auditory evoked potentials. To this end, we employed a conservative measure of amplitude contrasts without prior assumptions of regions of interest and followed a multilevel statistical approach (e.g., Henry and Obleser, 2012; Strauß et al., 2014). At the first level, we calculated independent-samples *t-*tests between the single-trial amplitude values of standards and deviants. Uncorrected by-participant *t*-values were obtained for all time-amplitude bins of all electrodes. At the second level, *t*-values were tested against 0 with dependent-sample *t-*tests. Taking into consideration the problem of multiple comparisons, a Monte-Carlo nonparametric permutation method with 1,000 randomizations, as implemented in fieldtrip (Oostenveld et al., 2011), estimated type I-error controlled cluster significance probabilities (at *p* < 0.05). In an electrode × time cluster (with Fz, Cz, CPz, between 130 and 200 ms post vowel onset), deviants elicited a significantly more negative response than standards (see Figure 3).

To analyze the EEG data for directional asymmetries, we calculated the iMMN as difference waves (deviant minus standard of the same words) in this aforementioned time window. Then, we calculated repeated-measures and Bonferroni corrected ANOVAs for each contrast with factors *word* (e.g., *Ziel* vs. *Zahl*) and *electrode* (Fz, Cz, CPz). Electrodes were chosen by cluster statistics.

In the /i:/—/a:/ contrast, we found both main effects for word (*F*_(1,16)_ = 7.286, *p* = 0.016) with a larger MMN for *Zahl* (*M* = −1,721, SEM = 0.36; *Ziel*: *M* = −0.586, SEM = 0.253) and for electrode (*F*_(2,32)_ = 14.634, *p* < 0.001) with strongest effect at Cz (*F*_(1,16)_ = 5.890, *p* < 0.05). In the vowel contrast /e:/—/a:/, there was not only a highly significant main effect for electrode (*F*_(2,32)_ = 12.307, *p* < 0.001) but also an interaction word × electrode (*F*_(2,32)_ = 4.942, *p* = 0.013). *Post hoc* analysis of the interaction showed a significant effect on Cz (*F*_(1,16)_ = 5.039, *p* < 0.05). Hence, we found asymmetries in visual inspection as well as in statistical analysis in both contrasts. The comparison of /u:/—/y:/ only revealed a main effect of factor electrode (*F*_(2,32)_ = 12.349, *p* < 0.001) with a marginal effect on CPz (*F*_(1,16)_ = 4.265, *p* = 0.055). Therefore, comparing *Süd* and *Sud*, we found an asymmetry in the visual inspection, which did not hold out statistical analysis. Hence, statistically we found a symmetrical effect.

The vowel contrast /i:/—/e:/ shows a symmetrical pattern in visual inspection and statistics with both main effects insignificant (word: *F*_(1,16)_ = 1.687, *p* = 0.212, electrode: *F*_(2,32)_ = 2.367, *p* = 0.110). The same is also true for the comparison of /i:/—/u:/ (word: *F*_(1,16)_ = 0.294, *p* = 0.595, electrode: *F*_(2,32)_ = 0.725, *p* = 0.492).

### Discussion

In summary, we found no clear evidence for neural asymmetries due to underspecification (FUL) but evidence for vowel discrimination based on phonetic salience of referent vowels (NRV). Furthermore, there were asymmetric as well as symmetric patterns in the MMN.

The asymmetric pattern of the comparison between /e:/—/a:/ was in line with the hypothesis of NRV. Here, /a:/ is a referent vowel in addition to being more peripheral, and discrimination of /e:/—/a:/ is, therefore, easier and comes with a stronger MMN effect than vice versa. Additionally, the symmetric effect in the contrast /y:/—/u:/ can also be explained with this model since both vowels are referents within this framework. The same holds good for the comparison of MMN effects between presentation orders of /i:/—/u:/. But in the latter contrast, there could be also a phonological explanation within the underspecification approach. The phonological variation in morphological processes can lead to different specifications of segments within words and therefore to effects that are at first sight not compatible within the FUL paradigm (Lawyer and Corina, 2018). The same is true for German umlauting back vowels. In our case, when deriving the plural of the German word *Stuhl*, the stem vowel is umlauting and fronting (*Stühle*). It can be assumed that umlaut is only possible if the stem vowel /u:/ is not specified for the place of articulation features and is therefore underspecified for backness (Scharinger, 2009; Scharinger et al., 2010). If the stem vowel of *Stuhl* is underspecified for the place of articulation information, asymmetry has to occur when it is compared to /i:/, which is also underspecified.

Contrary to this, the results of the remaining two contrasts are somewhat challenging since none of the previous operationalized models can explain the effects given in the data. Comparing the presentation orders in the contrast /i:/—/e:/, an asymmetric MMN pattern occurred. This is challenging the predictions of FUL as well as NRV since both models predict an asymmetry. According to the underspecification approach, MMN effects for *Steg* should be stronger (underspecification of mid vowel height), while NRV predicted that neural effects for *Stieg* should be stronger (/i:/ should act as focal referent here). An explanation for the symmetric effect could lie in the close phonetic distance of the vowels involved. There has been evidence from previous MMN studies that effects diminished or failed due to only small acoustic deviances in speech stimuli (Pettigrew et al., 2004a, b).

The most challenging results were obtained in the vowel contrast /i:/—/a:/. Although there is an asymmetric effect, both models failed to predict the direction of the found asymmetry: FUL predicted stronger effects for *Ziel* as coronal deviant due to mismatching place of articulation (PoA) information. In comparison with *Zahl* as standard, which is classified as a dorsal vowel (Scharinger, 2009), the extracted feature [COR] from the acoustic signal of /i:/ should evoke a mismatching stronger MMN. Also, the mismatching height features of those two vowels cannot have evoked the asymmetry. Since both height features ([HIGH] and [LOW]) involved are specified in the underlying representation, a mismatch occurs regardless of the presentation order. Since a mismatch of those features occurs in both presentation orders, they should not evoke an asymmetric effect. Additionally, the NRV model cannot explain the found asymmetric pattern either. According to NRV, asymmetry should have occurred since both vowels act as focal referents within this framework. The explanation for these results is still unclear. We argue that since the more abstract feature representations are based on acoustic properties (mainly formants), the effects could be more driven by changes in the acoustics than in feature representations. Because this is the contrast with the largest difference in terms of F1 or degree of openness, spectral characteristics (e.g., changes in F1) of the vowels could have been more involved in eliciting the surprising effects on an automatic and preattentive level. Additionally, changes in vowel quality do not only lead to changes of formants but also result in changes in other perceptual and psychoacoustic parameters. There is evidence that, for example, the perceived loudness of speech stimuli varies for vowel quality. That is, lower front vowels are perceived louder despite equal intensity (Glave and Rietveld, 1975, 1979) and vocal effort (Eriksson and Traunmüller, 1999, 2002). Thus, we hypothesize that psychoacoustic and perceptual parameters such as perceived loudness could have played a crucial role. This possibility is explored in greater detail using multiple regression analyses in “Explorative Analysis for Additional Influential Factors in MMN and log RT Data” section.

## Experiment 2: Reaction Times

Since our MMN results present some evidence that effects were not only driven by phonemic factors but also by acoustic differences, we decided to conduct a RT study in an attended listening task. It has been shown that the MMN evoked by unattended processing is sensitive to a great variety of different dimensions between standard and deviant. Here, preattentive processing has been proven to be sensitive also for low-level information like variations in duration and intensity (Näätänen et al., 1989; Paavilainen et al., 1991; Schröger, 1996; Jacobsen et al., 2003) or acoustic distance of stimuli (Savela et al., 2003; Deguchi et al., 2010). Since this component is highly sensitive to small low-level information differences (i.e., changes in frequency), higher-order information (for example phonemic identity) may be ignored or overridden in preattentive processing, for example, by acoustic proximity (Pettigrew et al., 2004a). Therefore, RT in an active discrimination task might reflect more cognitive, decision-based processing in which higher and more abstract effects like phonemic discrimination might surface better and more clearly. For this study, we thus propose the same hypotheses regarding potential asymmetries for both models as in Experiment 1.

### Participants and Materials

Twenty-six participants (17 females, mean age 24.43, SD 4.23) were recruited, all of whom were graduate or undergraduate students at Johannes Gutenberg University Mainz. They received monetary compensation for their efforts. All participants were right-handed monolingual German speakers with no active dialect competence and were socialized with Standard German. No participant reported neurological, psychological, or hearing impairments. Written informed consent was obtained from each participant before the experiment.

The stimuli used in Experiment 2 were the same as in Experiment 1. In contrast to the prior experiment, we had to reduce the number of tested vowel contrasts in order to shorten the session length (approximately testing 45 min). Therefore, we tested only the vowel contrasts /i:/—/a:/, /i:/—/e:/, and /i:/—/u:/. Contrasts were chosen as followed: /i:/—/e:/ and /i:/—/u:/ obtained in the MMN investigation symmetrical patterns and /i:/—/a:/ evoked an asymmetrical pattern. The symmetrical pattern of /i:/—/u:/ could be explainable with NRV and will therefore serve as control contrast for the remaining two vowel oppositions. Here, the iMMN results were not explainable by either of the models.

### Task and Procedure

Stimuli were presented in an active oddball setup, in which participants had to press a button as soon as they perceived the deviant. They were told to perform a categorical, phonemic decision (and therefore ignoring the inter-token variability; Johnson, 2015). During the experiment, subjects were seated comfortably in front of a screen in a sound shielded chamber. Sounds were presented with the Presentation software (version 16.4)^1^ at a comfortable volume *via* two loudspeakers to the left and right of the screen. The volume was set prior to the experiment and was kept equal across all subjects. All written instructions were presented on screen. This way, participants were also informed about the beginning and end of the experiment as well as pauses. Each contrast direction contained 180 standards and 20 deviants divided into two blocks. In total, 12 blocks were presented. Stimuli within blocks were randomized with 4–11 standards between two deviants. Blocks of the same condition never followed each other.

### Analysis and Results

The reaction time analysis was based on correct responses only (98% of data points included). RT were corrected for the onset cluster of each stimulus. Thus, measurement of RT began on the vowel onset. RT faster than 100 ms and slower than 1,000 ms were excluded. The remaining data were log-transformed to obtain an approximately normal distribution (Ratcliff, 1993; Whelan, 2008). Outliers (±2.5 SD) were removed before statistical analysis.

**Figure 4:**
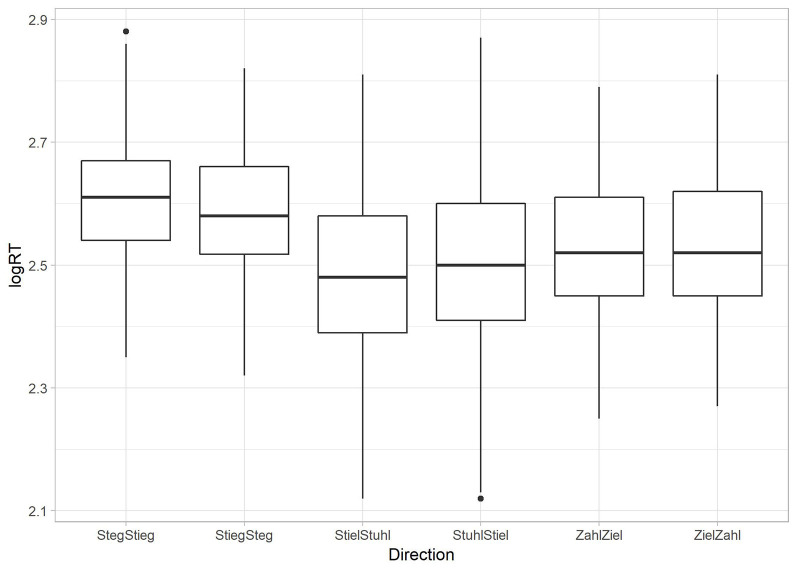
Reaction time results per condition. Reaction time results are given as log values per presentation direction of words with whiskers indicating the variance of the data and small dots representing outliers (but not extreme values) which were beneath the ±2 SD cut-off.

A repeated measures ANOVA with the factor *word* (e.g., *Ziel* vs. *Zahl*), controlled for multiple testing by applying Bonferroni correction, was calculated to reveal possible behavioral asymmetries. Here, we found a highly significant main effect (*F*_(5,2,320)_ = 107.811, *p* < 0.001). *Post hoc* analysis revealed asymmetric patterns in two of the tested vowel contrasts. /i:/—/e:/ was significantly faster than vice versa (*F*_(1,464)_ = 22.234, *p* < 0.001). The same was true for /i:/—/u:/ (*F*_(1,464)_ = 13.550, *p* < 0.001). However, in the vowel contrast /i:/—/a:/ a symmetrical pattern of RT occurred. Here, the *post hoc* analysis shows no difference between presentation directions (*F*_(1,464)_ = 0.793, *p* = 0.374; see Figure 4).

### Discussion

The behavioral study aimed to investigate the basis for some of the electrophysiological found effects in Experiment 1. There, both models could not provide a comprehensive explanation for our results, meaning that both models failed to explain all found effects. The vowel contrasts /i:/—/a:/ and /i:/—/e:/ were particularly challenging. Therefore, we conducted a reaction time experiment in an active oddball paradigm to investigate the previously found neural patterns in more detail. Overall, the RT indicate that in an active discrimination paradigm, German natural word stimuli were discriminated phonemically, based on higher-order abstract phonological features.

The RT for the vowel contrast /i:/—/e:/ and the observed asymmetrical pattern match with the predictions of FUL. The faster RT obtained when /e:/ was deviant seem to be due to the underspecification of [COR]. In this contrast, abstract representations may help to discriminate concerning the close phonetic distance of the vowels. There is evidence from an fMRI study indicating that participants had to rely more on abstract feature representations while discriminating acoustically very close vowels (Scharinger et al., 2016).

In contrast, the directional asymmetry of the vowel contrasts /i:/—/u:/ is, on first sight, more challenging for the underspecification approach since, following the theory, RT should be faster when subjects are presented with a fully specified vowel (e.g., /u:/) followed by an underspecified vowel (e.g., /i:/). But concerning the present study, we found the opposite effect for the obtained RT. The hypothesis of NRV for this contrast seems equally unsuitable: it states that asymmetric effect should occur because both vowels are reference vowels. In the case of /u:/ as deviant, one possible explanation for our findings could be that the additional labial feature drives the stronger effect. This additivity would then “overwrite” the feature mismatch. Several studies proved that the MMN is sensitive to an additivity effect correlated with the amount of deviating dimensions (Schröger, 1995; Takegata et al., 1999, 2001a,b; Wolff and Schröger, 2001). Similar observations have been made in an fMRI study in which an increasing number of features led to stronger activation in the superior temporal sulcus (STS). Besides., the effect of stronger STS activations was also seen in reaction time measures whereby reaction time decreased with increasing feature number (Scharinger et al., 2016). Furthermore, in a MEG study, it was shown that N1m amplitudes increased when feature number increased (Scharinger et al., 2011a). More evidence for the additive effect has been brought to light in a MEG study with consonants, in which labial, specified glides produced stronger MMFs than coronal glides (Scharinger et al., 2011b). Under the assumption of an additivity effect of the phonological feature [LAB], we argue that the underspecification approach still holds since this model predicts effects based on sparse and abstract phonological features.

For the symmetrical effect in the contrast /i:/—/a:/, there are two explanations we believe to be conceivable. The first one is that the hypothesis of NRV holds good. Since both vowels of this contrast are reference vowels in the framework, there is no discriminatory advantage in either direction. But why participants rely on phonetic features in these cases remains a question. The second more likely explanation argues within the underspecification approach: we classified Standard German /a:/ as a dorsal vowel. But there is articulatory evidence (see Figure 1), evidence from theoretical analysis (Wiese, 2000), and also neurobiological evidence (Obleser et al., 2004) that Standard German /a:/ is likely not specified for a place of articulation. Thus, there is no place feature mismatch anymore for /i:/ and /a:/. Since the remaining height features [LOW] and [HIGH] are both specified and mismatching regardless of the presentation order, asymmetry has to occur.

In conclusion, it seems that participants use phonological and phonemic cues in vowel discrimination within natural German words. But the effects found in Experiments 1 and 2 are different although the same experimental paradigm has been applied. The reason for different effect patterns in the electrophysiological and behavioral data could lie in the different attention requirements or differences of involved processing levels between the two tasks, but to this point, it is still not clear.

## Explorative Analysis for Additional Influential Factors in Mmn and Log Rt Data

Because the interpretation of the MMN and RT data with common models is challenging, and because both models failed to explain the found patterns comprehensively, we decided to test for additional influential factors in both datasets.

Vowel perception could be influenced not only by vowel identity but also by acoustic properties like intensity, duration, and fundamental frequency (Näätänen et al., 1989; Paavilainen et al., 1991; Schröger, 1996; Jacobsen et al., 2003; Peter et al., 2010; Pakarinen et al., 2013). For most of these factors, researchers commonly try to exclude or control in the stimuli preparation procedure, but some acoustic factors cannot be avoided. For instance, since the phonological feature oppositions to distinguish different vowel qualities (i.e., high vowels vs. low vowels) are based on formants (Lahiri and Reetz, 2010), they also automatically imply an acoustic difference. Moreover, when words are used as stimuli, lexical features like frequency of occurrence (Alexandrov et al., 2011; Shtyrov et al., 2011) or phonotactic probability (Bonte et al., 2005; Yasin, 2007; Emmendorfer et al., 2020) are known to interfere in speech perception and vowel discrimination. Especially in our approach, where we tested the hypotheses of the models by using natural German spoken words, those influences may contribute to patterns of results. Therefore, even though we here focused on the identity MMN, i.e., on electrophysiological responses to physically identical stimuli in different conditions, we decided to test whether and which of these factors have an influence on our electrophysiological and which affected the behavioral data. For this purpose, we operationalized different acoustic, phonological, and lexical factors. Furthermore, we also took acoustic and perceptual factors beyond the well-known ones (e.g., degree of openness and perceived loudness) into account to disentangle their contribution to the iMMN and RT data patterns. This in-depth analysis is explorative and has never been done this extensively before.

### Preparation: Rating of Implicit Loudness

One possible additional influence beyond the well-known factors could be the perceived loudness of the stimuli. Here, loudness is referring to the magnitude of the auditory sensation (Fletcher and Munson, 1933; not the physical intensity), but has been mainly taken as a perceptual correlate for sound intensity. Note that it has been shown that the physical intensity of sounds and perceived loudness are measures on different auditory dimensions. While physical intensity is stimuli-inherent, the perceived loudness of stimuli is a perceptual phenomenon and therefore subject-dependent (Yanushevskaya et al., 2013). Moreover, while perceived loudness and sound intensity might be expected to be treated as equal, hearing research showed that two sounds of the same intensity can be rated with different perceived loudness levels due to various factors (e.g., spectral characteristics, bandwidth; Moore, 2003). Additionally, perceived loudness levels could be correlated to gender differences since there is evidence that females perceive sounds louder than males despite the same sound pressure level (Hamamura and Iwamiya, 2016). Furthermore, there is evidence from sound processing that cortical activations are more likely driven by perceptual factors (e.g., perceived loudness) than physical characteristics (e.g., physical intensity; Langers et al., 2007). Therefore, it could be possible in our study, although the stimuli words were normalized for the same average intensity and vowel intensity was approximately the same across words, that participants perceived the two words in a minimal pair as strongly different in terms of perceptual or sensational loudness.

#### Materials, Subjects, and Procedure

To test the word stimuli of Experiments 1 and 2 for differences in the perceived (or implicit) loudness, we conducted a rating study. Here, 10 subjects (seven females, three males) participated, all of the students or employees at Johannes Gutenberg-University of Mainz who reported normal hearing. They were all monolingual German speakers (with a mean age of 32.6 years, SD 9.6) and gave written consent before the rating.

The word stimuli were arranged in the same minimal pairs as in the previous experiments. We tested all five minimal pairs in both presentation orders. Because, we had five tokens per word in each presentation order, there were 25 possible combinations per presentation order, resulting in 250 trials overall. The trials were randomly arranged in ten blocks with 25 trials per block. Block order differed between subjects. The study was conducted *via* Presentation (version 16.4)^1^, and auditory stimuli were delivered *via* headphones on the same listening level for all participants. For the experiment, all participants were seated in a quiet room.

At the beginning of the experiment, the instructions were presented on the computer screen. Afterward, each trial started with a 1,500 ms blank screen. Following a fixation star to keep participants engaged with the experiment, both words were then presented (ISI: 800 ms). After the presentation of the second word, a short blank screen (600 ms) was presented before two question marks with a timeout of 2,500 ms appeared. The question marks were used as an indication for participants to give their answer *via* button press. Participants were instructed to rate the perceived loudness of the two words of each minimal pair in comparison to one another. Three answers were possible: first word louder, second word louder, or both words equally loud.

#### Analysis and Results

**Figure 5:**
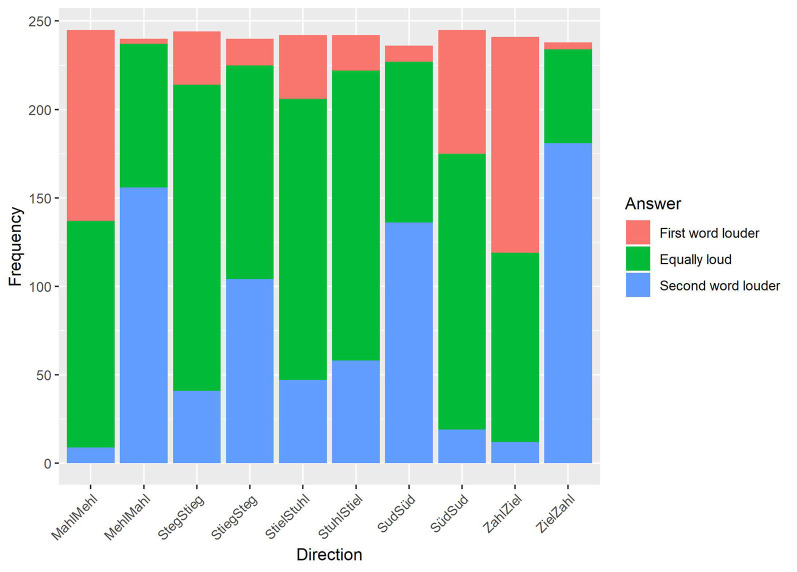
Results of the perceived loudness rating. The results are plotted for each presentation direction (*x*-axis) in relation to the frequency of the given responses (*y*-axis).

Having collected the responses of all participants, frequency values of the three answer categories (first, second, and equal) for each minimal pair per presentation order were calculated. Timeouts were not included in the analysis. The distributions of answers for each direction can be seen in Figure 5. The Pearson chi-square test, calculated in IBM SSPS (version 21) with variables *direction* (10) and *answer* (3), showed that the relationship between both variables is highly significant ($\chi_{\left(\text{18}\right)}^{\text{2}}$ = 998.986, *p* < 0.001).

In preparation for the operationalization of the factor of implicit loudness for the multiple regression analysis, frequency values, transformed in percentage with the highest given answer, will be taken into account in the next step of the analysis. The percentages of answers per direction are given in Table 3.

**Table 3:** Distribution of the answers for the perceived loudness of words (in percent) with the most given answer per presentation order in bold.

Direction/condition	Equal	First	Second
Mahl—Mehl	**52.2**	44.1	3.7
Mehl—Mahl	33.8	1.3	**65.0**
Steg—Stieg	**70.9**	12.3	16.8
Stieg—Steg	**50.4**	6.3	43.3
Stiel—Stuhl	**65.7**	14.9	19.4
Stuhl—Stiel	**67.8**	8.3	24.0
Sud—Süd	38.6	3.8	**57.6**
Süd—Sud	**63.7**	28.6	7.8
Zahl—Ziel	44.4	**50.6**	5.0
Ziel—Zahl	22.3	1.7	**76.1**

The descriptive results are indicating a possible influence on the MMN data: in the contrast /e:/—/a:/ (Mahl—Mehl, Mehl—Mahl), participants rated the word pair as equally loud by a higher percentage if *Mehl* was the second word (52.2%). In the reverse direction, the second presented word *Mahl* was more likely perceived as louder than *Mehl* (65%). Since, in this contrast, the MMN effect of *Mahl* (as deviant) was greater than in the reverse direction, implicit loudness could have a potential influence on the preattentive processing of words.

In the contrast /i:/—/a:/ (Zahl—Ziel, Ziel—Zahl), the word *Zahl* was perceived more often as louder regardless of the presentation order. In the first presentation order (Zahl—Ziel), *Zahl* was perceived in 50.6% as louder, and in the reverse direction, with *Zahl* as the second word, it was also rated as louder (76.1%). The neural data showed clear MMN effects in both directions, with an asymmetric, because stronger, the result for *Zahl* as deviant. It may be that the higher implicit loudness of *Zahl* has driven (Ziel—Zahl) or reduced (Zahl—Ziel) the neural effects.

In the next contrast with words *Sud* and *Süd*, similar patterns to the first one can be observed. When *Süd* was presented as the second word, participants perceived it as being louder (57.6%). When *Süd* was presented as the first word, both words were rated more often as equally loud (63.7%). Taking the MMN results into account, it might again be possible that implicit loudness affected the neural data. While the MMN effects are statistically symmetric, there is a slightly stronger effect for *Süd* as deviant (than in the reverse direction), when the plotted data are inspected.

In the last two contrasts (*Steg* and *Stieg*, *Stuhl* and *Stiel*), both words were described more often as equally loud within both presentation orders (Steg—Stieg: 70.9%, Stieg—Steg: 50.4%; Stiel—Stuhl: 65.7%, Stuhl—Stiel: 67.8%). Since the MMN data showed a symmetrical pattern in the statistical analysis, it could be stated that the perceived loudness might have influenced the neural effects once more.

Additionally, and following the feedback of participants, it can be hypothesized that implicit loudness could be correlated with the degree of openness of the long vowels. Especially with larger openness differences between vowels (/i:/—/a:/, /e:/—/a:/), the more open vowel /a:/ was rated as louder than the closer counterparts. Regarding the openness difference of /i:/ and /e:/, it can be stated that, phonetically, the difference here is smaller than between /i:/—/a:/ and /e:/—/a:/, and therefore the loudness effect could be perceptually reduced or inhibited. Moreover, the different MMN results, despite equal loudness rating patterns of /e:/—/a:/ (asymmetric MMN) and /y:/—/u:/ (symmetric MMN), could also support the hypothesis that the perceived loudness is correlated with the degree of openness because of the latter contrast’s lack of height difference. Contrary, the first-mentioned contrast differs in vowel height and openness, and therefore the influence of perceived loudness could lead to stronger neural effects.

### Explorative Analysis *via* Multiple Regressions

Because of the challenging and unexpected electrophysiological effects that do not match the behavioral results in addition to the descriptive identification of a potential influence of the implicit loudness, we decided to also investigate the possible influences of several additional factors on the neural (iMMN difference values of mean voltages between standard and deviant of the same stimulus) as well as the behavioral level (log RTs).

#### Defining Factors

Fifteen potential influential factors were defined, based on theoretical (*specificity, peripherality, focality*) and empirical input (*implicit loudness, electrodes, degree of openness*) as well as stimulus-inherent characteristics (*F1, F2, F3, frequency of words, bigram frequency, f0, vowel duration, intensity*). Additionally, one control factor (*contrast*) has been taken into account. All factors were operationalized and calculated with mean values per word category (i.e., mean F1 for *Mahl*) since the iMMN data as well as log RT data were also obtained as averaged data (for example in iMMN data, all tokens for *Mahl* as deviant or standard are collapsed in respectively one mean amplitude value).

The theoretical factors of *specificity* and *peripherality* have been operationalized concerning both evaluated models in Experiments 1 and 2. While *specificity* (difference value between the number abstract features in the deviant minus the number of features in the standard) refers to FUL and takes the additivity effect discussed in the RT data into account, *peripherality* has been operationalized according to the assumption of NRV that peripheral vowels may be more salient and act as referents in vowel discrimination as a categorical variable (discrimination towards a more peripheral vowel, towards a more central vowel, or no referent/equal position in the vowel space). Additionally, *focality* has been operationalized according to the notion in NRV that the universal preference of referent vowels may be alternated in adults due to language experience and is therefore taking the formant convergences in our stimulus set into account. Focality was again operationalized as a categorical variable (discrimination from a less to a more focal vowel or from a more focal to a less focal vowel).

The empirically motivating factors have been chosen from the input of the loudness rating. As mentioned before, the results of the rating study suggest an influence of *implicit loudness* on the neural data. Therefore, this factor has been included in the further analysis as a function of the answer category with the highest percentage per presentation order (first word louder, second word louder, equally loud). Because there could be a possible relationship between loudness and the openness of vowels, the *degree of openness* (increasing openness of the mouth, decreasing openness, equal openness) was also taken into account.

Since, we tested natural (and mostly unmanipulated) German words in this study, there were stimuli-inherent differences between words that were controlled but could not be excluded in the preparation of the stimuli. The three first factors of this category are the differences between standard and deviant in terms of a change in *F1, F2, and F3*. To display the presentation orders of the stimuli in this factor, difference values (e.g., mean F1 of deviant minus mean F1 of standard) were calculated. Another possible stimulus-inherent influence is the *frequency* of occurrence of the words used. Since, we wanted to test a large set of long vowels, we had to choose monosyllabic minimal pairs to reduce testing time. Being restricted by the German lexicon, we were not able to perfectly balance the lexical frequency of words; therefore, there are frequency differences between the words making up the minimal pairs. To test the influence of the *frequency* of occurrence on the electrophysiological and behavioral patterns, we included this factor in the explorative analysis in the form of difference values (log lexical frequency of the deviant minus log lexical frequency standard). The same was true for the factor *bigram frequency* (bigram frequency deviant minus bigram frequency standard). The same holds good for the factors *f0, vowel duration*, and *intensity*. To evaluate the influence of the stimuli-inherent differences in fundamental frequency, vowel duration, and intensity, we operationalized those factors also as difference values between deviant and standard (*f0*: the difference between mean f0 of the deviant minus mean f0 of the standard; *vowel duration*: the difference between vowel duration of the deviant minus the vowel duration of the standard; *intensity*: the difference between mean vowel intensity of the deviant minus mean vowel intensity of the standard).

Last but not least, the *contrast* has been included as a controlling factor, since the iMMN and RT effects were different for the tested vowel oppositions. Here, both presentation orders of each minimal pair are combined.

#### Correlation and Single Linear Regressions

In preparation for the multiple regression analysis, we conducted first Kendall’s Tau correlation for all previously defined factors. Additionally, we calculated for each factor a single regression model on the MMN data to identify reasonable factors to be included in the final multiple regression analysis.

Correlation analysis showed very strong correlation between the factors *F2* and *specificity* (τ = −0.907, *p* < 0.001) and *F1* and *degree of openness* (τ = 0.866, *p* < 0.001), *vowel duration* and *degree of openness* (τ = 0.856, *p* < 0.001), as well as *F2* and *F3* (τ = 0.867, *p* < 0.001). Because of that, we only included *specificity*, *F1*, and *F3* as theoretically implied factors. Additionally, *vowel* *duration* was included in the analysis since there is evidence that sound duration is influencing the perceived loudness (Todd and Michie, 2000). The exclusion of the other factors was necessary to avoid collinearities.

Additionally, strong, but in respect with collinearity uncritical correlations, were found between the following factors: *F1* and *f0* (τ = −0.764, *p* < 0.001), *F3* and *specificity* (τ = −0.760, *p* < 0.001), *F1* and *vowel duration* (τ = 0.764, *p* < 0.001), *vowel duration* and *f0* (τ = −0.778, *p* < 0.001), *specificity* and *peripherality* (τ = 0.559, *p* < 0.001), *bigram frequency* and *peripherality* (τ = 0.676, *p* < 0.001), *vowel duration* and *intensity* (τ = −0.689, *p* < 0.001), *f0* and *intensity* (τ = 0.556, *p* < 0.001), *focality* and *F1* (τ = −0.603, *p* < 0.001), *F2* (τ = 0.507, *p* < 0.001) as well as F3 (τ = 0.686, *p* < 0.001), *focality* and *f0* (τ = 0.686, *p* < 0.001), *focality* and *vowel duration* (τ = −0.745, *p* < 0.001), with *intensity* (τ = 0.566, *p* < 0.001), with *degree of openness* (τ = −0.731, *p* < 0.001) and *implicit* *loudness* (τ = −0.654, *p* < 0.001), *implicit loudness* and *f0* (τ = −0.775, *p* < 0.001), *implicit loudness* and *F1* (τ = 0.667, *p* < 0.001), *implicit loudness* and *vowel duration* (τ = 0.660, *p* < 0.001), *implicit loudness* and *degree of openness* (τ = 0.603, *p* < 0.001), and *implicit loudness* and *intensity* (τ = −0.488, *p* < 0.001).

Single factor regression (with MMN data) revealed the influence of only five significant factors with reasonable *R*^2^ and adjusted *R*^2^ values: *implicit loudness* (Δ*R*^2^ = 0.023, *p* < 0.001), *contrast* (Δ*R*^2^ = 0.019, *p* < 0.001), *intensity* (Δ*R*^2^ = 0.016, *p* < 0.001), *f0* (Δ*R*^2^ = 0.012, *p* ≤ 0.001), *vowel duration* (Δ*R*^2^ = 0.013, *p* < 0.001).

#### Hierarchical Multiple Regressions for MMN and Log RT Data

The five previously identified factors were applied to hierarchical multiple regression with separate calculations for the MMN and the RT datasets in five steps with the following order: implicit loudness (model 1), contrast (model 2), f0 (model 3), vowel duration (model 4), and intensity (Model 5).

**Figure 6:**
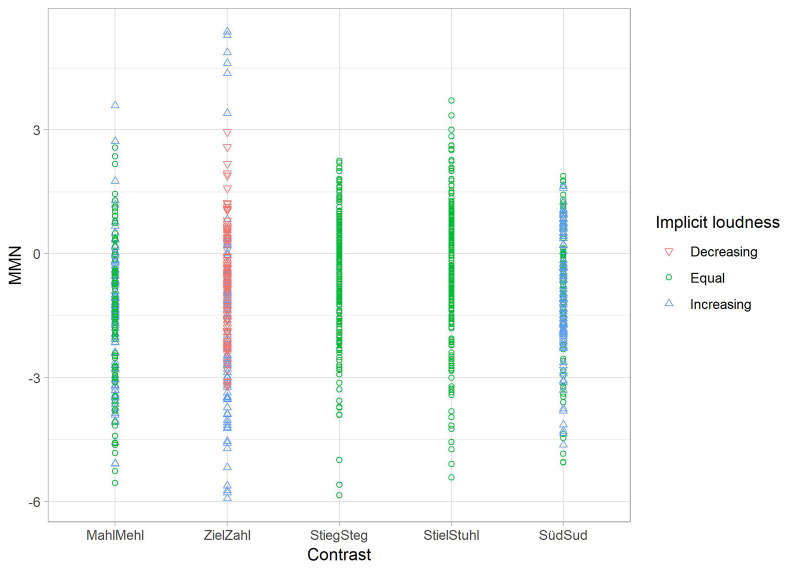
Scatterplot for the regression analysis of the given iMMN data from Experiment 1. MMN difference values of each participant (*y*-axis) per vowel contrast (*x*-axis) in relation to implicit loudness. Increasing loudness (deviant louder than standard) is shown as a blue triangle, decreasing loudness (standard louder than deviant) as a red triangle, and equal perceived loudness as a green dot. MMN difference values are scaled with perceiving the stimuli as equally loud (most clearly seen in vowel contrasts StiegSteg and StielStuhl).

Results for the MMN dataset, for which the relevant key figures are displayed in Table 4, indicate that only the first two factors (implicit loudness and vowel contrast) are contributing to account for variance and that implicit loudness and contrast are approximately an equal fit for the explanation of results. In this study, implicit loudness seems to influence the neural results of the MMN study (see Figure 6).

**Table 4:** Multiple regression models for the MMN dataset.

Model#		b	SE B	β	*p*
1	Constant	−0.363 (−0.592, −0.133)	0.117		0.002
	Loudness	−0.436 (−0.607, −0.265)	0.087	−0.155	0.000
*R*^2^ = 0.024, Δ*R*^2^ = 0.023, *p* < 0.001					
2	Constant	−0.703 (−0.971, −0.434)	0.137		0.000
	Loudness	−0.436 (−0.605, −0.267)	0.086	−0.155	0.002
	Contrast	0.170 (0.098, 0.242)	0.037	0.143	0.000
*R*^2^ = 0.044, Δ*R*^2^ = 0.043, *p* < 0.001					

**Figure 7:**
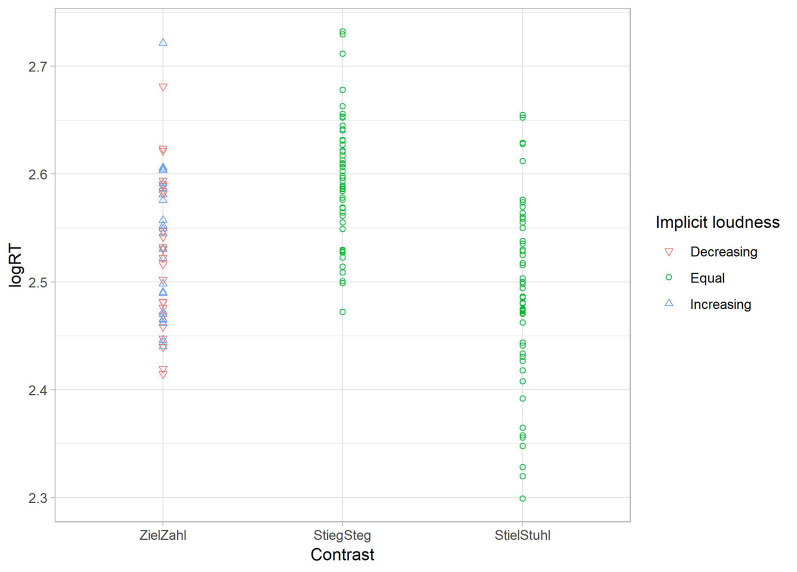
Scatterplot for the regression of the obtained reaction time (RT) data from Experiment 2. Mean log RTs of each subject (*y*-axis) are depicted per vowel contrast (*x*-axis) in relation to implicit loudness. RT results (log-values) are not scaled by the perceived loudness of the stimuli.

In contrast, results of the second multiple regression model in the log RT dataset implicate that the factors influencing the neural results are not contributing to the explanation of behavioral patterns. Once again, Model 2 (implicit loudness and contrast) is fitting best with contrast as the only significant contributing regression coefficient (Table 5). Thus, implicit loudness is not contributing to the found behavioral pattern in the reaction time experiment (see Figure 7).

**Table 5:** Multiple regression models for the reaction time (RT) dataset (n.s. = no significance).

Model#		b	SE B	β	*p*
1	Constant	2.547 (2.522,2.573)	0.013		0.000
	Loudness	0.001 (−0.021,0.024)	0.011	0.010	n.s.
*R*^2^ = 0.000, Δ*R*^2^ = −0.006, n.s.					
2	Constant	2.558 (2.535,2.595)	0.015		0.000
	Loudness	0.001 (−0.020,0.023)	0.011	0.127	n.s.
	Contrast	−0.021 (−0.033′6, −0.005)	0.008	−0.204	0.011
*R*^2^ = 0.041, Δ*R*^2^ = 0.029, *p* < 0.05					

### Discussion

Multiple regression analysis on both datasets revealed that the perceptual factor of perceived loudness had only an influence on neural effects. Here, it can be stated that regarding the MMN data, the phonological and phonetic status of the vowels presented in the minimal pairs played a role in the elicitation of MMN effects (factor contrast), but—crucially—effects were simultaneously driven by the implicit loudness of the presented words. Overall, it seems that the neural effect was scaled with perceiving the stimuli as equally loud. Put differently: the more strongly one word was perceived as being louder, the further the MMN difference values deviated from zero. Therefore, it can be argued that perceived, or implicit, loudness seems to be an important factor in interpreting the found neural patterns.

This is especially true for those contrasts for which both models (NRV and FUL) failed to explain the effects. Especially in the symmetrical contrasts of *Stieg—Steg* and *Stiel—Stuhl*, implicit loudness seems to drive the symmetry as in this contrast, both words were more often perceived as equally loud regardless of the presentation order. The small visible (but statistically not significant effect) in the contrast *Süd—Sud* could also be explained by this factor since there were more increasing judgments if *Süd* was the second word. Missing statistical significance could be a result of the correlation of loudness with a degree of openness. Since this contrast did not differ in terms of vowel height (and therefore openness), the influence of perceived loudness could be weaker than in vowel oppositions with height differences. Turning to the asymmetrical patterns in the MMN data, it can be stated that for the pattern of *Ziel—Zahl*, which was especially challenging because both models could not explain the found asymmetry, implicit loudness once more seems to drive the neural effects since *Zahl* was more often perceived as louder regardless of the presentation order. If *Zahl* served as deviant, the greater perceived loudness has led to a stronger effect than in the reverse direction. Here, the greater implicit loudness of the standard could have reduced MMN effects. In the last contrast (i.e., *Mehl—Mahl)*, differences in perceived loudness and degree of openness could have led to the stronger effect for *Mahl* (as deviant). *Mahl* as the second presented word was perceived as louder than in the reverse direction. In this direction, the degree of openness is also increasing. In the reverse direction, equally perceived loudness might have elicited smaller effects.

Turning to the RT data, multiple regression analysis revealed that the perceived loudness did not influence the behavioral patterns; therefore, the effects are more likely driven by the traditional models (namely FUL and NRV) discussed before.

## General Discussion and Conclusion

In this article, we reported the results of an electrophysiological and a behavioral study as well as an explorative analysis of influential factors on vowel discrimination. While most studies investigating speech sound discrimination only tested two or three vowel oppositions, we conducted our MMN study on a much larger stimulus set. Here, we investigated preattentive vowel processing with five different vowel contrasts covering the most important German long vowels embedded in real and natural German words. To obtain an even more natural listening situation, we used five tokens per word, which resulted in a large stimulus set. The purpose of the investigations reported here was 2-fold: first, we wanted to compare two often discussed models for vowel discrimination to investigate which model can explain the found effects in German in the best way. Second, we wanted to shed further light on factors influencing vowel discrimination on the neural and behavioral levels. For this purpose, we conducted an in-depth analysis delving into possible confounds to a degree that has not been investigated so far.

To summarize the results of the electrophysiological experiment concerning the first research question, we found MMN evidence for discrimination and perceptual asymmetries (or symmetries) in vowel perception according to the NRV model. Three contrasts showed facilitated and asymmetric discrimination on presenting a less peripheral vowel as standard and a more peripheral vowel as deviant. These results are in line with other behavioral and electrophysiological studies (e.g., Masapollo et al., 2015, 2017b; Zhao et al., 2019) that also report easier discrimination from a more central to a more peripheral vowel. Only one contrast could be explained within the underspecification approach. By contrast, both models failed to explain the found symmetric neural patterns. For those vowel oppositions, it could be possible that the phonemic discrimination was overridden in preattentive processing through acoustic proximity (Pettigrew et al., 2004b) or sensational interferences caused by perceived loudness. The lack of phonemic discrimination and weighting of sensational influences in the MMN experiment could be due to experimental protocol since the subjects were not instructed to perform phonemic discrimination, but to ignore the stimulation (Johnson, 2015). To test those challenging results, we investigated these contrasts with a behavioral active oddball paradigm and instructed participants here to perform a phonemic decision. Thus, we assumed that in the active oddball paradigm subjects had to activate more abstract mental representation more strongly due to allophonic variance in the stimuli; therefore blending out simple acoustic differences in the decision making. The behavioral results, in contrast to the MMN effects, showed that participants were able of phonemic discrimination based on abstract representations. Here, the found patterns can only be fully explained by the underspecification approach, in line with previous studies delivering evidence for speech sound discrimination with the help of sparse and abstract features (e.g., Eulitz and Lahiri, 2004; Lahiri and Reetz, 2010; Scharinger et al., 2012a, b).

In summary, the results of Experiments 1 and 2 are challenging in two ways. First, the neural pattern cannot be explained comprehensively by either of the two models. Second, the neural and behavioral patterns do not match. The lack of compliance between electrophysiological and behavioral results can be interpreted in terms of an attention shift and cue weighting as a function of task dependency. Differences in cue weighting due to attention shifts have been reported in several studies. Szymanski et al. (1999) conducted an MMN study with and without attention on the stimulation and found differences in the neural responses. They interpreted these findings in terms of a modulation of the memory trace in the attended condition. Here, attention leads to the activation of more accurate and precise representations of the standards, which in turn generate larger responses of the deviant. Therefore, it can be argued that in attended stimulation, more information is accessible for discrimination than in unattended conditions. Similar results concerning the richness of mental representation accessible during discrimination as a function of attention were also found by Tuomainen et al. (2013). In an MMN study with an active go/no-go task, they found that in the attentive task participants were able to use more spectral attributes in vowel discrimination than when they listened passively to the stimulation. The authors interpreted the results as a change in perceptual discrimination strategy due to the attention shift. Furthermore, Savela et al. (2003) found, in a study combining MMN (passive oddball) and RT (active oddball), that subjects discriminated the used Finnish and Komi vowels differently depending on the task. While the behavioral results indicated phonemic discrimination of the vowels, the preattentive MMN patterns were more driven by acoustic differences than phonemic representations. Concerning our electrophysiological and behavioral results, it can be assumed that the attention shift between the passive and the active oddball has led to differences in cue weighting in vowel discrimination. We argue that in the active experiment, participants were able to discriminate phonemically, which led to patterns explainable by common models. In contrast to this—but following previous studies—the passive MMN patterns are based not only on phonemic but also on acoustic or perceptual differences.

To address this issue further, we conducted an explorative analysis of influencing factors of the electrophysiological as well the behavioral datasets. We included several theoretical, lexical, and phonotactic factors that are known to influence results. While other studies found an influence on neural data, for example, of phonotactic probabilities (Bonte et al., 2005; Yasin, 2007; Emmendorfer et al., 2020) or the lexical frequency of words (Alexandrov et al., 2011; Shtyrov et al., 2011; Aleksandrov et al., 2017), we cannot provide evidence for those factors neither on the electrophysiological nor on the behavioral data. On the contrary, we have identified a new influencing factor on MMN data: we found that neural effects were not only driven by phonemic features but also by the perceptual and psychoacoustic differences in perceived loudness in the stimuli. In contrast, no such influence of this factor could be found in the behavioral data. Therefore, the multiple regression analyses on both datasets support the aforementioned interpretation on different discrimination strategies since we found that the influence of perceived loudness of the word stimuli only mattered in the neural but not the behavioral data. Once again, these results can be interpreted as evidence that in preattentive processing, more perceptual and acoustic features are responsible for the elicitation of effects. But when attention was shifted towards the stimulation (like in the active oddball paradigm of the RT experiment), these perceptual factors receded into the background, and discrimination was based on phonemic representations of the perceived vowels only.

Although perceived loudness is related to (and heavily determined by) sound intensity, two sounds of equal perceived loudness may well have different levels of sound intensity (Yanushevskaya et al., 2013). This is due to the processing of auditory stimuli in the cochlea (Moore, 2003), which depends not only on the characteristics of the stimuli, such as bandwidth but on the listener as well. We found evidence that speech signals of approximately equal intensity could still be perceived to be of different loudness. Additionally, we could show that within our datasets, perceived loudness was highly positively correlated with the degree of openness of the vowels and changes in F1. We conclude that perceived loudness differences could be guided by differences in the degree of openness since with increasing openness of the tested vowels, the perceived loudness of words increased as well, and since increasing loudness elicited larger MMN effects. These results add evidence to the hypothesis that perceived loudness of vowel stimuli is also linked to vowel quality (Glave and Rietveld, 1975, 1979). Additionally, we found correlations of perceived loudness and changes in f0, intensity, and vowel duration. But the multiple regression analysis showed that those additional factors did not contribute to the found neural asymmetries. Here, only the differences in perceived loudness can explain the found patterns. However, since there is evidence that perceived loudness can be influenced by vowel duration (Todd and Michie, 2000) and changes in fundamental frequency (Hsu et al., 2015), more studies are needed to disentangle all the factors contributing to differences in the sensational perceived loudness of stimuli and influencing natural vowel processing.

To our knowledge, we are the first to find evidence for the influence of perceived loudness on the perception of German long vowels and MMN data regarding natural vowel processing. We propose that the perceptual, or implicit, the loudness of stimuli can act as an intermediate representation level between stimuli-inherent acoustics and abstract phonological features. The exact influence of perceptual and psychoacoustic factors, like perceived loudness in speech processing, is still underinvestigated and more research is needed. But for the time being, our results provide evidence that studies should include more factors beyond the well-known (and theoretically driven) when analyzing and interpreting neural and behavioral data.

## Data Availability Statement

The raw data supporting the conclusions of this article will be made available by the authors, without undue reservation.

## Ethics Statement

The studies involving human participants were reviewed and approved by Ethics Committee of the Society of German Linguistics (DGfS). The patients/participants provided their written informed consent to participate in this study.

## Author Contributions

MR contributed to the design of the work, acquisition, analysis, and interpretation of the data, as well as drafting of the work. AW contributed to the design of the work, acquisition, and revising the manuscript. AN contributed to the design of the work, acquisition and analysis of the data, as well as the revising of the manuscript. MS contributed to the design of the work, analysis and interpretation of the data, as well as drafting and revising of the manuscript. All authors gave final approval of the version to be submitted and agreed to be accountable for all aspects of the work ensuring that questions related to the accuracy or integrity of any part of the work are appropriately investigated and resolved. All authors contributed to the article and approved the submitted version.

## Conflict of Interest

The authors declare that the research was conducted in the absence of any commercial or financial relationships that could be construed as a potential conflict of interest.

## Footnotes

^1^http://www.neurobs.com
